# Overexpression of microRNA-107 suppressed proliferation, migration, invasion, and the PI3K/Akt signaling pathway and induced apoptosis by targeting Nin one binding (NOB1) protein in a hypopharyngeal squamous cell carcinoma cell line (FaDu)

**DOI:** 10.1080/21655979.2022.2051266

**Published:** 2022-03-16

**Authors:** Xin Gao, Xinlong Fan, Wei Zeng, Jiwang Liang, Nan Guo, Xiao Yang, Yuejiao Zhao

**Affiliations:** Department of Head and Neck Surgery, Cancer Hospital of China Medical University, Liaoning Cancer Hospital & Institute, Shenyang, People’s Republic of China

**Keywords:** Hypopharyngeal squamous cell carcinoma, miR-107, cell behavior, PI3K/Akt, NOB1

## Abstract

Hypopharyngeal squamous cell carcinoma (HSCC) is one of the most common head and neck cancers, with a worst prognosis owing to its aggressivity. MicroRNA-107 (miR-107) is reported to regulate the progression of various cancers. Nevertheless, its implied function in HSCC remains unclear. This study is aimed to exploring the roles and potential mechanisms of miR-107 in HSCC. We found that miR-107 expression was significantly decreased in HSCC tissues compared with the para-cancer tissues. Moreover, miR-107 overexpression by miR-107 mimics decreased FaDu cell viability, led to cell cycle arrest in G1/S phase, accelerated apoptosis, and reduced cell migration and invasion. MiR-107 possibly resulted in deactivation of the phosphatidylinositol 3-kinase (PI3K)/Akt pathway, evidenced by the decrease of phosphorylated (p-) PI3K and p-Akt. Besides, dual-luciferase reporter assay confirmed that miR-107 might bind to the 3’UTR of Nin one binding protein 1 (NOB1), and elevated NOB1 expression in HSCC tissues and a negative correlation between miR-107 and NOB1 were found. Rescue assays demonstrated the significant roles of miR-107 in FaDu cell behavior by modulating NOB1. In addition, the tumorigenic potential of miR-107 *in vivo* was conducted. It was found that miR-107 overexpression in FaDu cells significantly inhibited tumor growth and led to inactivation of the PI3K/Akt signaling. The above findings revealed that miR-107 could suppress FaDu cell proliferation, migration, invasion and induced apoptosis by targeting NOB1 through the PI3K/Akt pathway, suggesting that miR-107/NOB1 axis may exert a key role in FaDu HSCC development.

## Graphical abstract

**Figure:**
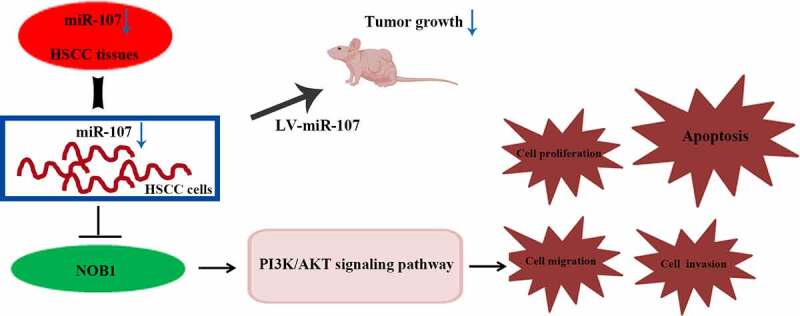

## Introduction

Hypopharyngeal squamous cell carcinoma (HSCC), a common type of aggressive head and neck squamous cell carcinoma (HNSCC), is often found at an advanced stage accompanied by a poor prognosis and a 5-year survival rate of 25–40% [1–3]. In spite of valuable measures taken such as surgery, radiotherapy, and chemotherapy for curing HSCC, rare improvement in survival has been achieved [4]. As such, it is important for seeking more effective therapeutic options to attenuate the onset of HSCC.

MicroRNAs (miRNAs) are conserved single-stranded non-coding RNA molecules that exhibit essential roles in the occurrence and development of many human cancers [5]. For example, miR-140-5p inhibits migration and invasion of HSCC cells as evidenced by Jing et al [6]. In addition, miR-107 expression is down-regulated in head and neck cancers including laryngeal cancer, esophageal cancer, as well as tongue squamous cell carcinoma [7–10]. Clinical and bioinformatics studies indicate low expression of miR-107 in HPV-positive oropharyngeal cancer, and show that miR-107 is highly correlated with the overall survival of this type of tumor [11,12]. MiR-107 plays a key part in oropharyngeal squamous cell carcinoma via regulation of multiple target genes and signaling pathways [12]. Besides, miR-107 is involved in the regulation of malignant biological behaviors in Wilms’ tumor, including apoptosis, migration and invasion [13]. The research by Xia et al. reveals that miR-107 suppresses tumor development through modulating brain-derived neurotrophic factor and PI3K/Akt pathway in A549 cells [14]. Nevertheless, the effect of miR-107 on HSCC has not been discovered at present.

Bioinformatics analysis reveals that a target gene of miR-107 may be Nin one binding protein 1 (NOB1), which has been widely considered as a transcriptional modulator [15]. A growing body of evidence indicates that NOB1 may be related to a series of tumors including papillary thyroid carcinoma, oral squamous cell carcinoma, and colorectal cancer, and it may act as an oncogenic factor [16–18]. Furthermore, the high level of NOB1 in ovarian cancer is discovered by Lin et al [19]. Gao and colleagues point out that knockdown of NOB1 repressed Hep2 cell growth and metastasis [20]. However, the role of NOB1 in HSCC remains unclarified.

In the current study, we aimed to investigate the function of miR-107 and its underlying mechanism in FaDu HSCC. It was shown that miR-107 suppressed FaDu cell proliferation, migration and invasion, and promoted apoptosis, accompanied by activation of the PI3K/Akt signaling pathway, which may be mediated by the down-regulation of NOB1. The findings signify that miR-107 may be a promising target for monitoring FaDu HSCC.

## Materials and methods

### Human samples

A total of 30 HSCC tissue samples and 30 matched nontumorous adjacent tissues were obtained from patients who underwent surgery at Cancer Hospital of China Medical University, Liaoning Cancer Hospital & Institute. All experiments were performed under the supervision of the Ethical Committee of the Cancer Hospital of China Medical University, Liaoning Cancer Hospital & Institute and in line with Declaration of Helsinki. All patients signed the informed consent forms.

### Cell culture and transfection

HSCC cell line FaDu (Procell, China) was cultured in MEM (M0643, Sigma, USA) containing 10% fetal bovine serum (FBS, F8067, Sigma, USA) in a humidified atmosphere (37°C, 5% CO_2_). On the one hand, cells were transfected with miR-107 mimics or mimics negative control (NC) or the NOB1 overexpressing plasmid or the empty vector. On the other hand, cells were co-transfected with miR-107 mimics or mimics NC and the NOB1 overexpressing plasmid or the empty vector. All transfection experiments were performed using Lipofectamine 2000 (11668–019, Invitrogen, USA) in accordance with the instructions of the manufacturer.

### Cell counting kit-8 (CCK-8) assay

CCK-8 assay was carried out to measure cell viability at 24 h or 48 h or 72 h of transfection. In brief, FaDu cells were cultured with CCK-8 solution (C0037, Beyotime, China). After 1 h of incubation at 37°C, the optical density value at 450 nm was recorded [21].

### Flow cytometer (FCM) analysis

After transfection for 48 h, the cell cycle detection kit (KGA512, KeyGEN, China) and the apoptosis detection kit (KGA106, KeyGEN) were used to measure cell cycle distribution and apoptosis, respectively [22]. The cells used to detect cell cycle were washed with PBS and collected by centrifugation (310 g, 5 min). They were then fixed with 70% ethanol overnight at 4°C, rinsed, and suspended in 0.5 mL solution including propidium iodide (PI) and RNaseA (PI:RNaseA = 9:1). After incubation for 45 min at room temperature (RT), they were analyzed by FCM (C6, BD Sciences, USA).

The cells used to detect cell apoptosis were resuspended. Then, the Annexin V-FITC solution (5 μL) was added to cells and mixed, and then PI (5 μL) was added. After reaction at RT for 10 min in the dark, the apoptosis rate was assessed via FCM.

### Wound-healing assay

Cells were scratched with a 200 μL pipette tip, and then incubated (37°C, 5% CO_2_) in serum-free medium for 48 h. After that, the average distance of cell migration was recorded under a 100 × microscope (IX53, Olympus, Japan) [23].

### Transwell assay

Cell invasion assay was performed utilizing the chambers of 24-well Transwell plates (3422, Corning, USA). Briefly, subsequent to coating 40 μL of Matrigel (356234, BD Sciences) diluted with serum-free medium at 1:3 on ice, the upper and lower chambers were filled with 200 cell suspension (1.5 × 10^4^/well) and 800 μL of medium containing FBS, respectively. After 24 h of incubation (37°C, 5% CO_2_), transwell chambers were fixed for 20 min at RT, followed by staining with 0.1% crystal violet (0528, Amresco, China). At last, the number of invading cells in each group was recorded under a microscope at 200 × magnification [23].

### Dual luciferase reporter assay

HEK 293 T cells were co-transfected miR-107 mimics or mimics NC and NOB1 3’ untranslated region (3’-UTR) wild type (WT) or NOB1 3’-UTR mutant type (MT) for 48 h. After that, the relative luciferase activity was determined by the corresponding kit (E1910, Promega, USA) [24].

### In vivo *tumor formation experiment*

All animal experiments in this study were approved by the standards of Care and Use enacted by Laboratory Animals of Cancer Hospital of China Medical University, Liaoning Cancer Hospital & Institute. Six-week-old nude mice were adaptively fed for a week under controlled living conditions. They were randomly divided into lentivirus (LV)-NC group and LV-miR-107 group. FaDu cells (4 × 10^6^ cells) with LV-based miR-107 or LV-NC transfection were subcutaneously injected into the right armpit of the mice. Once the tumor was macroscopic, the tumor volume was measured every 3 days. At day 24, tumor tissues were collected and weighted. Meanwhile, tumor tissues were partly stored at −70°C and partly fixed for subsequent experiments [25].

### Hematoxylin and eosin (H&E) staining

The fixed tissues were dehydrated, paraffin-embedded, and sectioned into five-μm thickness. The sections were dewaxed and dehydrated. After staining with hematoxylin and eosin, the pathological changes of sections were discovered using a light microscope (× 200 magnification).

### Immunohistochemistry (IHC) assay

After deparaffinage and dehydration, the sections were cultured with boiling antigen retrieval solution for 10 min and cultured with 3% H_2_O_2_ at RT for 15 min. Then, sections were rinsed, incubated with goat serum (SL038, Solarbio) at RT, and incubated with anti-NOB1 (DF12216, dilution 1:50, Affinity Biosciences, China) overnight at 4°C. Next, they were cultured with HRP-labeled goat anti-rabbit IgG (#31460, dilution 1:500, Thermo Scientific, USA) at RT for 1 h. Ultimately, the sections were stained with 3,3’-diaminobenzidine (DAB, DA1010, Solarbio), counterstained with hematoxylin for 3 min at RT, and visualized under a microscope at 400 × magnification.

### Real-time quantity PCR (RT-qPCR)

Total RNA extraction was performed using the RNA extraction kit (RP1201, BioTeke Corporation, China) in accordance to the standard protocols. The complementary DNA (cDNA) was generated using the miRNA First Strand cDNA Synthesis (Tailing Reaction) kit (#B532451, Sangon Corporation, China; for miRNA) or M-MLV reverse transcriptase (for mRNA). MiR-107 and NOB1 levels were quantified by TaqMan RT-qPCR using SYBR Green (EP1602, BioTeke Corporation). The primers utilized in RT-qPCR were from Genscript Biotech (China) as follows: miR-107 (5’-AGCAGCATTGTACAGGGCTATCA-3’, forward); NOB1 (5’-GTGGGAACAAGACCCTGAA-3’, forward; 5”-GGGAGTGGGAAGCGAGTA-3’, reverse); β-actin (5’-GGCACCCAGCACAATGAA-3’, forward; 5’-TAGAAGCATTTGCGGTGG-3’, reverse).

### Western blot analysis

Total protein were extracted utilizing the lysis buffer (P0013, Beyotime) containing 1% PMSF (ST506, Beyotime). The concentration was evaluated by BCA protein assay kit (P0011, Beyotime). Forty μg of protein was separated on sodium dodecyl sulfate-polyacrylamide gel electrophoresis (SDS-PAGE) and then transferred onto PVDF membranes. After blocking with 5% nonfat milk, the membranes were incubated with primary antibodies (dilution 1:1000) overnight at 4°C, and cultured with secondary antibodies (dilution 1:5000) for 1 h at 37°C. Then the blots were detected by the ECL kit (P0018, Beyotime). The information of antibodies used in immunoblotting was as below: cyclin D1 (A19038), cyclin-dependent kinase 4 (CDK4, A0366), caspase-3 (A19654), bcl-2 associated X (bax, A19684), bcl-2 (A0208), E-cadherin (A11492), vimentin (A19607) all from ABclonal Technology (China); matrix metalloproteinase (MMP)-2 (10373-2-AP) and MMP-9 (10375-2-AP) both from Proteintech Group, Inc. (China); phosphorylated (p-) PI3K (Tyr358, AF3242), PI3K (AF6241), p-Akt (Ser473, AF0016), Akt (AF6261), and NOB1 (DF12216) all from Affinity Biosciences; β-actin (sc-4778) from Santa Cruz (USA); goat anti-rabbit IgG (A0208) and goat anti-mouse IgG (A0216) both from Beyotime.

## Statistical analysis

Measurement data were indicated as means ± SD. Comparisons of data among multiple groups were conducted with one-way analysis of variance (ANOVA), using GraphPad Prism 8.0. The unpaired Student t test was used to assess the difference between the two groups. *P* < 0.05 indicates statistical significance.

## Results

### Expression of miR-107 was aberrantly down-regulated in HSCC tissues

**Figure 1.**
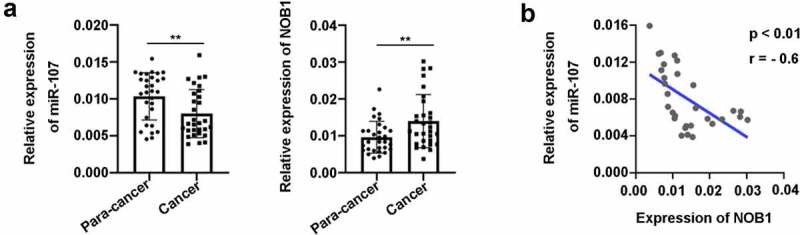
MiR-107 expression was aberrantly down-regulated in HSCC tissues. (a) The relative expression of miR-107 and NOB1 was determined by RT-qPCR assay in HSCC tissues (n = 30) versus para-cancer tissues (n = 30). (b) An inverse expression correlation between miR-107 and NOB1 in HSCC. Data were shown as means ± SD. ***P < 0.001 versus para-cancer group.

To investigate the biological function of miR-107 in HSCC, firstly, RT-qPCR was carried out to determine miR-107 expression in 30 HSCC samples and 30 para-cancer tissues. The results showed that the relative expression of miR-107 was significantly down-regulated in HSCC (Figure 1(a)), indicating a potential suppressive role of miR-107 in HSCC. Moreover, NOB1 level was up-regulated in HSCC tissues (Figure 1(a)), and NOB1 expression was inversely associated with miR-107 level in HSCC tissues (Figure 1(b)), indicating that miR-107 and NOB1 may be associated in the regulation of HSCC.

### Upregulated miR-107 inhibits FaDu cell proliferation

**Figure 2.**
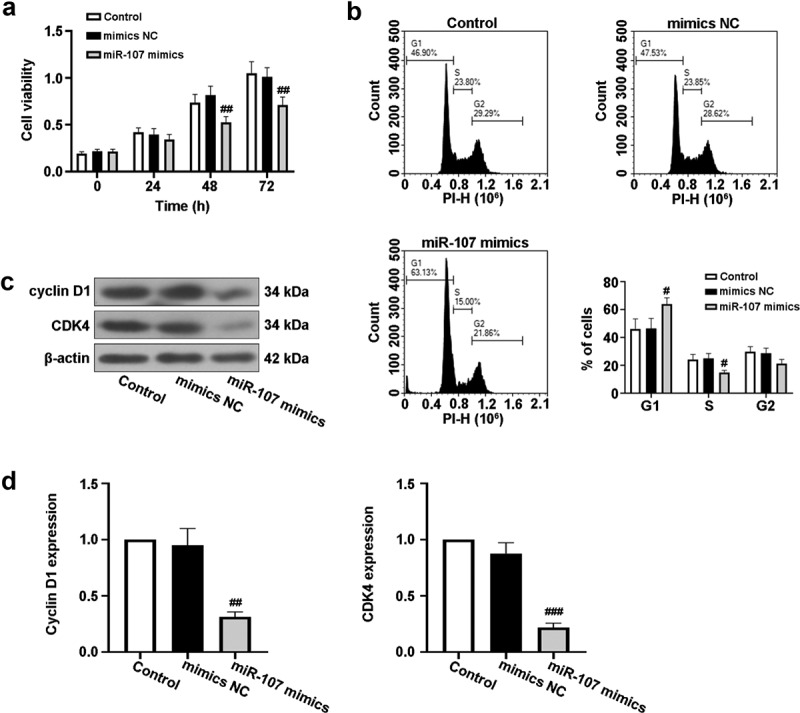
MiR-107 overexpression inhibited proliferation and arrested cell cycle in FaDu cells. FaDu cells were transfected with miR-107 mimics or mimics negative control (NC). (a) After transfection for 24 h or 48 h or 72 h, cell viability was detected by CCK-8 assay. (b) After transfection for 48 h, cell cycle was evaluated via flow cytometer (FCM). (c, d) After transfection for 48 h, the protein expressions of cyclin D1 and CDK4 were assessed using Western blot. Results were presented as means ± SD (n = 3). ^#^*P* < 0.05 and ^##^*P* < 0.01 versus the mimics NC group.

To expound the role of miR-107 in HSCC, FaDu cell line was selected and miR-107 overexpression was achieved by miR-107 mimics transfection, as shown in Figure S1(a). Following that, we discovered that cell viability was obviously lowered by miR-107 mimics at 48 h and 72 h after transfection (Figure 2(a)). Moreover, FCM results demonstrated that miR-107 overexpression increased the proportion of FaDu cells in G1 phase of the cell cycle, but reduced the proportion of cells in S phase (Figure 2(b)). In addition, the protein expressions of cyclin D1 and CDK4 related to cell cycle were assessed. Western blot results revealed that cyclin D1 and CDK4 levels were reduced by miR-107 overexpression (Figure 2(c,d)). These findings indicated that upregulated miR-107 lowered cell proliferation possibly through the arrest of cell cycle.

### MiR-107 overexpression increases apoptosis of FaDu cells

**Figure 3.**
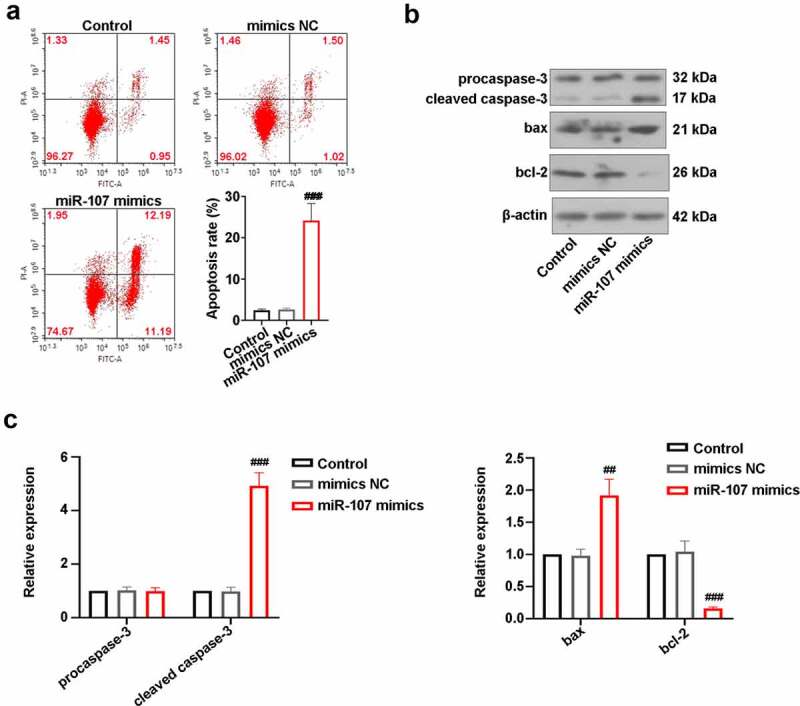
MiR-107 overexpression induced FaDu cell apoptosis. (a) After transfection for 48 h, FCM was utilized to detect apoptosis. (b, c) Measurement of caspase-3, bax, and bcl-2 protein levels. Data were expressed as means ± SD (n = 3). ^###^*P* < 0.001 versus the mimics NC group.

After transfection for 48 h, the apoptosis rate of FaDu cells was evaluated utilizing FCM. As displayed in Figure 3(a), there were no statistical differences in apoptosis rate between control cells and mimics NC-transfected cells. However, the apoptosis rate was obviously elevated in cells by miR-107 mimics transfection. Besides, the increase of cleaved caspase-3 and bax expressions and the decrease of blc-2 level were induced by miR-107 overexpression (Figure 3(b,c)). The above results manifested that miR-107 facilitated FaDu cell apoptosis.

### Upregulated miR-107 suppresses FaDu cell migration and invasion

**Figure 4.**
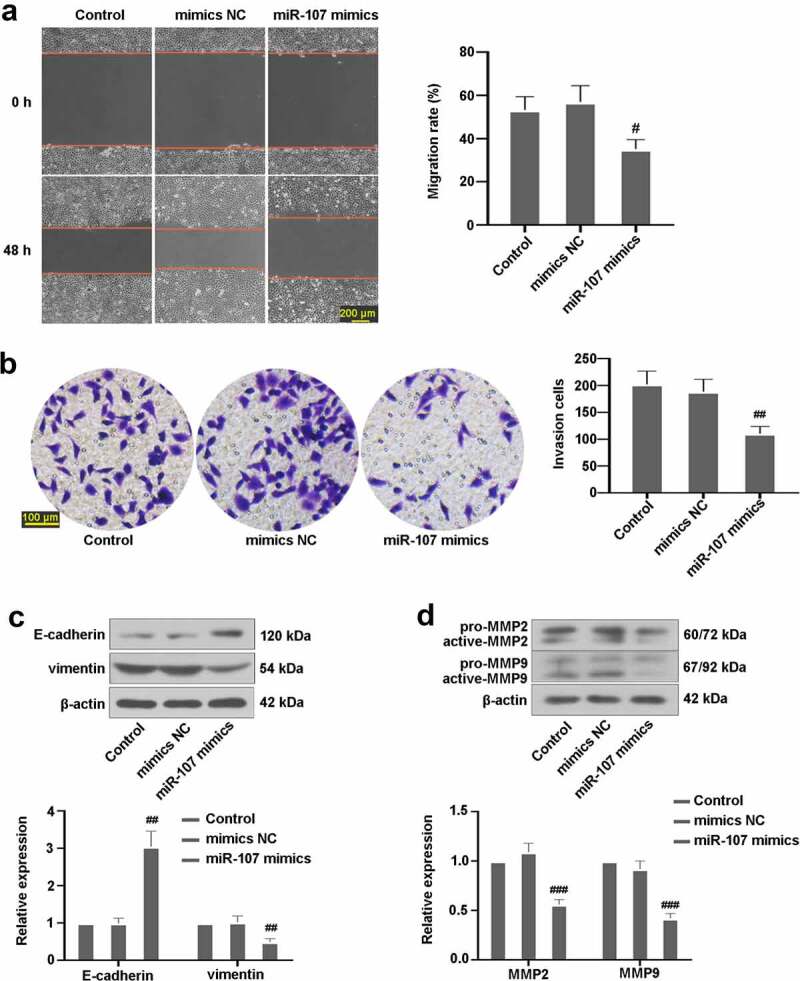
MiR-107 overexpression reduced FaDu cell migration and invasion. (a) Wound scratch assay in FaDu cells transfected with miR-107 mimics or mimics NC for 48 h was conducted. Scale bar = 200 μm. (b) Transwell assay in FaDu cells transfected with miR-107 mimics or mimics NC for 48 h was carried out. Scale bar = 100 μm. (c) Detection of E-cadherin and vimentin expressions. (d) Assessment of MMP2 and MMP9 levels. Results were presented as means ± SD (n = 3). ^#^*P* < 0.05 and ^##^*P* < 0.01 versus the mimics NC group.

After 48 h of incubation, whether miR-107 affected the migration and invasion of FaDu cells was explored. The findings showed that miR-107 upregulation led to a decrease in migration rate (Figure 4(a)). Besides, the number of invasion cells was markedly reduced in cells following miR-107 mimics transfection (Figure 4(b)). Furthermore, upregulated miR-107 not only increased the expression of E-cadherin, but also decreased vimentin, active-MMP2, and active-MMP9 levels (Figure 4(c,d)). These findings showed the inhibitory effects of miR-107 on FaDu cell migration and invasion.

### MiR-107 inhibited PI3K/Akt activation in FaDu

**Figure 5.**
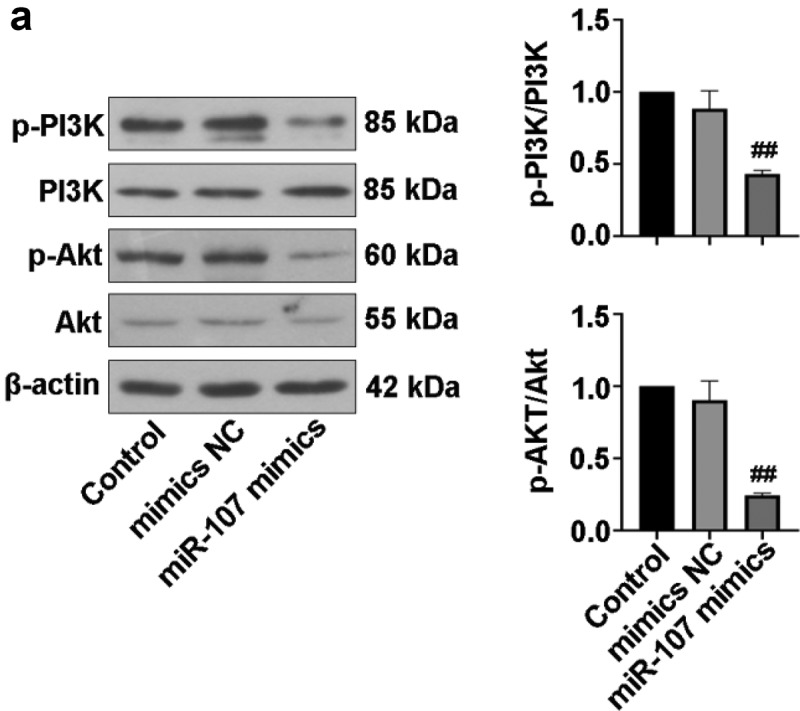
MiR-107 overexpression suppressed the PI3K/Akt signaling pathway in FaDu cells. (a) After transfection for 48 h, the protein levels of p-PI3K, PI3K, p-Akt, and Akt were evaluated.

Previous studies have revealed that PI3K/Akt signaling pathway is implicated in HSCC [26]. Here, expressions of proteins associated with PI3K/Akt pathway were examined. As exhibited in Figure 5(a), no remarkable changes in PI3K and Akt expressions were visualized in the three groups. The expressions of p-PI3K and p-Akt in cells with miR-107 mimics transfection were lower than those in mimics NC-transfected cells, suggesting that upregulated miR-107 possibly suppressed the PI3K/Akt signaling pathway.

### NOB1 binds to the 3’-UTR of miR-107

**Figure 6.**
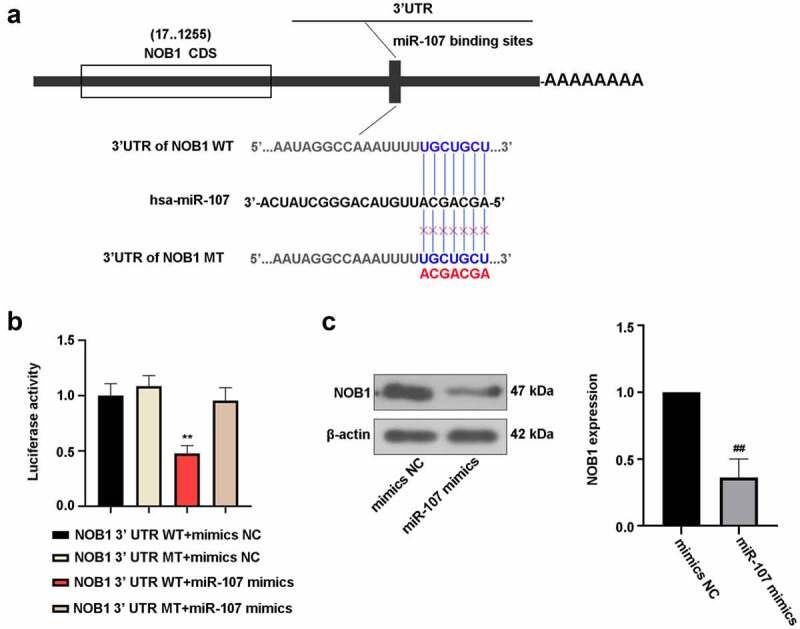
NOB1 was a target gene of miR-107. (a) MiR-107 target site in the NOB1 3’ UTR WT was predicted. (b) The dual luciferase reporter assay was employed to demonstrate that NOB1 was a target gene of miR-107. (c) FaDu cells were transfected with miR-107 mimics or mimics NC for 48 h, and the protein expression of NOB1 was detected. Data were expressed as means ± SD (n = 3). **P < 0.01 versus the NOB1 3’ UTR MT+miR-107 mimics group. WT, wild type; MT, mutant type.

The target relationship between miR-107 and NOB1 3’-UTR were predicted using bioinformatics website, and the possible binding sites of miR-107 and NOB1 WT or NOB1 MT were presented in Figure 6(a). To confirm this prediction, luciferase assay was conducted to assess the luciferase activity. The decrease of luciferase activity was observed in 293 T cells by co-transfecttion of miR-107 mimics and NOB1 WT as compared to miR-107 mimics and NOB1 MT-co-transfected cells (Figure 6(b)). Additionally, miR-107 overexpression significantly lowered NOB1 level (Figure 6(c)). It was concluded that miR-107 could target NOB1 3’-UTR and negatively regulate its expression.

### Effect of miR-107 on FaDu cells was abrogated by regulation of NOB1

After transfection with the NOB1 overexpressing plasmid or the empty vector for 48 h, the protein level of NOB1 was detected. As shown in Figure S1(b), NOB1 expression was up-regulated in the NOB1 overexpressing plasmid-transfected cells in comparison with the vector group, indicating high transfection efficiency.

**Figure 7.**
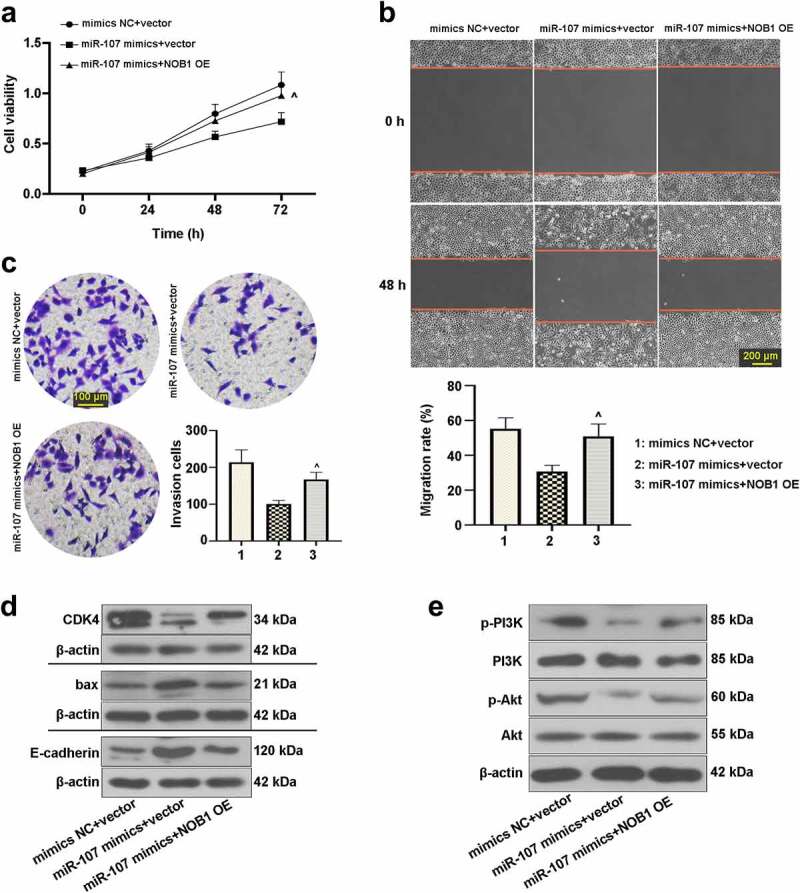
MiR-107 overexpression repressed cell proliferation, migration, invasion, and the PI3K/Akt signaling pathway by regulation of NOB1 in FaDu cells. Cells were co-transfected with miR-107 mimics or mimics NC and the NOB1 overexpressing plasmid or the empty vector. (a) After co-transfection for 24 h or 48 h or 72 h, CCK-8 assay was used to measure cell proliferation. (b, c) Migratory and invasive abilities of cells at 48 h post-transfection were evaluated. Wound-healing assay: scale bar = 200 μm; Transwell assay: scale bar = 100 μm. (d, e) After co-transfection for 48 h, CDK4, bax, E-cadherin, p-PI3K, PI3K, p-Akt, and Akt protein expressions were assessed. Results were presented as means ± SD (n = 3). ^*P* < 0.05 and ^^*P* < 0.01 versus the miR-107 mimics+vector group.

We further assessed whether miR-107 affects FaDu cell behaviors via modulating NOB1. After co-transfection with miR-107 mimics or mimics NC and the NOB1 overexpressing plasmid or the empty vector, we analyzed the function of miR-107 and NOB1 on FaDu cells. It was shown that NOB1 overexpression enhanced cell viability after transfection for 72 h (Figure 7(a)). As depicted in Figure 7(b,c), promotion of cell migrated and invasive abilities could be induced by upregulation of NOB1. Also, Figure 7(d,e) revealed that NOB1 overexpression increased CDK4, p-PI3K, and p-Akt expressions, but decreased the bax and E-cadherin. The above results further confirmed the interaction between miR-107 and NOB1 in FaDu cells, and the role of miR-107 was probably modulated by NOB1.

### *MiR-107 overexpression reduced tumor growth* in vivo

**Figure 8.**
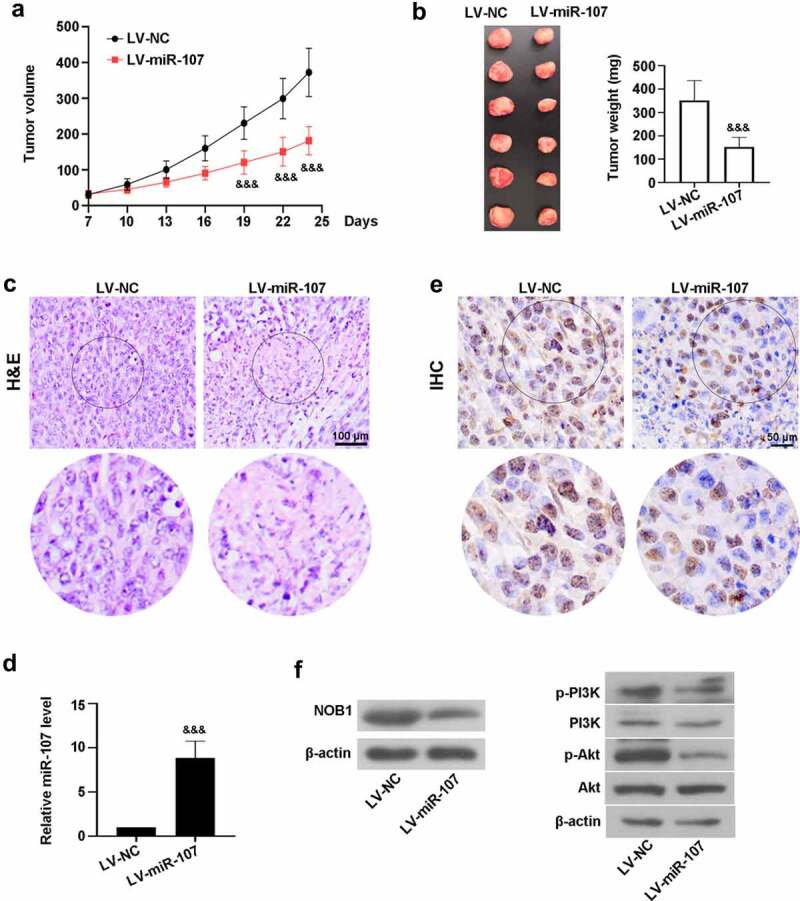
MiR-107 overexpression reduced the tumorigenic potential of FaDu HSCC *in vivo*. FaDu cells transfected with LV-based miR-107 or LV-NC were subcutaneously injected into the right armpit of the mice. (a) The tumor volume was measured every 3 days when the tumor was macroscopic. (b) At day 24, the tumor was collected and weighted. (c) The tumor tissues were stained with H&E. Scale bar = 100 μm. (d) The level of miR-107 was detected by RT-qPCR. (e) The expression of NOB1 was measured via IHC assay. Scale bar = 50 μm. (f) Evaluation of NOB1, p-PI3K, PI3K, p-Akt, and Akt protein expressions. Data were expressed as means ± SD (n = 6). ^&&&^*P* < 0.001 versus the LV-NC group. LV, lentivirus.

Furthermore, we investigated the role of miR-107 in tumorigenicity i*n vivo*. As shown in Figure 8(a), compared with the control mice transplanted with LV-NC-treated cells, the tumor volume was significantly diminished by miR-107 overexpression from day 19 to day 24. The weight of the tumors obviously decreased in the LV-miR-107 group at day 24 post-transplantation (Figure 8(b)). Besides, H&E staining uncovered that the tumor tissues from the LV-NC group showed obvious necrosis, while miR-107 could improve the histopathological changes (Figure 8(c)). High level of miR-107 was observed in mice transplanted with LV-miR-107-administrated cells (Figure 8(d)). IHC assay uncovered that miR-107 reduced NOB1 level (Figure 8(e)). Besides, the protein expressions of NOB1, p-PI3K, and p-Akt were down-regulated in mice via overexpression of miR-107 (Figure 8(f)). These data indicated that miR-107 overexpression lowered tumor growth, probably through suppressing NOB1 and PI3K/Akt signaling pathway.

## Discussion

It was the first time to discover the function of miR-107 on regulating the behaviors of HSCC FaDu cells. Here, we revealed that miR-107 expression was down-regulated in HSCC tissues. MiR-107 overexpression not only inhibited FaDu cell proliferation, migration and invasion, but also promoted apoptosis and deactivation of PI3K/Akt signaling pathway through *in vitro* experiments. It has become evident that miRNAs can control a series of cellular processes via combining with the 3’-UTR of target mRNAs, which causes their decrease or inhibition of translation [27]. Given this, we further verified the interaction between miR-107 and NOB1 in this study, and found that overexpressed miR-107 reduced NOB1 expression. In addition, rescue assays verified that the miR-107’s role in FaDu cells was modulated by NOB1. We also conducted the animal experiments to assess the function of miR-107 on tumor growth of HSCC. It was found that the overexpressed miR-107 reduced the tumorigenic potential, which was in line with Xia et al’s study [14].

The roles of miR-107 in different tumor growth and progression are distinguishing. One example is that miR-107 blocks Ewing sarcoma cell proliferation as evidenced by Chen et al [28]. MiR-107 was also found to reduce cell viability in melanoma [29]. On the contrary, another study by Liu et al. revealed that miR-107 facilitated cell cycle entry and suppressed apoptosis in colon cancer cells [30]. However, the function of miR-107 in HSCC cells has not been understood. In our current study, miR-107 reduced cell cycle entry of FaDu cells, which was consistent with the earlier literatures [31,32]. Additionally, dysregulated cell proliferation is usually concomitant with the changes of cell cycle. Herein, cell cycle distribution and the expressions of proteins associated with cell cycle were measured. It was demonstrated that miR-107 overexpression slowed cell cycle entry, along with the reduction of cyclin D1 and CDK4 expressions. Similar findings were found in the previous research that up-regulation of miR-107 arrested cell cycle distribution in lung cancer [14]. Apoptosis, a crucial event that affects cell growth, plays an important part in the tumor progression [20,33]. Once apoptosis occurs, cleaved caspase-3 and bax can be activated while bcl-2 is inactivated. Our present data suggested that overexpression of miR-107 promoted the apoptosis. In line with this result, knockdown of TBX3 enhanced the apoptosis of FaDu cells [34]. In addition, *in vivo*, we found that miR-107 overexpression reduced tumor volume and weight, and improved the histopathological changes, probably implying the anti-tumor role of miR-107 in FaDu HSCC. Overall, the experiments indicated that miR-107 repressed FaDu cell proliferation and facilitated apoptosis, and miR-107 may play an inhibitory role in tumor growth of FaDu HSCC.

Invasion and migration are the pivotal causes of tumor initiation. The miR-107 level was connected with cancer metastasis depending on cell types. For instance, the high level of miR-107 significantly reduced the abilities of migrated and invasive of SGC-7901 cells in gastric cancer [35]. Inhibition of miR-107 in pancreatic ductal adenocarcinoma cells (ASCP-1 and PANC-1) also lowered cell invasion [36]. Herein, the overexpression of miR-107 significantly was found to inhibit FaDu cell migration and invasion, consistent with a previous report [10]. Moreover, cell adhesion molecule E-cadherin acts as a prognostic factor of HPCC [34]. Active MMP2 and active MMP9 are biomarkers of invasion in tumors. As a result, miR-107 resulted in the upregulation of E-cadherin expression, and the downregulation of vimentin as well as active MMP2 and MMP9, further demonstrating the inhibitory roles of miR-107 in FaDu cell invasion and migration.

The PI3K/Akt pathway is a vital signal transduction pathway that regulates multiple cellular functions in various cancers [14,26]. In this signaling pathway, PI3K serves as an upstream effector, probably mediating the activation of Akt via phosphorylation of serine residues [37]. Hyperactivation of PI3K/Akt pathway is a wide tumor driver, which is closely involved in the occurrence and development of HSCC, ovarian cancer, and breast cancer [^38–40^]. Nevertheless, the effect of miR-107 on PI3K/Akt in FaDu cells is still unknown. In this study, the expressions of the pathway-related proteins were determined. The findings showed that the decrease of p-PI3K and p-Akt was induced by miR-107 overexpression, thus possibly inhibited the activation of this signaling pathway. Besides, it has been documented that MAPK pathway is intimately linked to renal cancer cell proliferation and HSCC cell growth and migration [41,42]. Gao et al. confirmed that inhibition of NOB1 (a target gene of miR-107) lowered cell development in laryngeal cancer, arrested cell cycle, and induced apoptosis by regulating the MAPK/JNK signaling pathway [20]. As such, it is worthy of further studying the function of miR-107 overexpression on the JNK pathway in FaDu cells in future.

## Conclusion

In summary, our research indicated that miR-107 up-regulation reduced FaDu cell proliferation, migration, invasion, and the activation of PI3K/Akt signaling pathway, and facilitated apoptosis possibly through modulation of NOB1. Also, miR-107 delayed the growth of FaDu cell xenograft tumor. Therefore, miR-107 may be a novel target for the treatment of FaDu HSCC.

## Figures and Tables

**Table:** 

miRNAs	microRNAs
3’UTR	3’ untranslated region
CCK-8	Cell counting Kit-8
H&E	hematoxylin and eosin
RT-qPCR	quantitative reverse transcription-PCR
PI3K	phosphatidylinositol 3-kinase
HSCC	hypopharyngeal squamous cell carcinoma

## Data Availability

The authors confirm that the data supporting the findings of this study are available within the article.
